# Toward autonomous event-based sensorimotor control with supervised gait learning and obstacle avoidance for robot navigation

**DOI:** 10.3389/fnins.2025.1492436

**Published:** 2025-02-25

**Authors:** Shahin Hashemkhani, Vijay Shankaran Vivekanand, Samarth Chopra, Rajkumar Kubendran

**Affiliations:** Department of Electrical and Computer Engineering (ECE), Swanson School of Engineering, University of Pittsburgh, Pittsburgh, PA, United States

**Keywords:** sensorimotor control, central pattern generator, dynamic state machine, multi-timescale feedback, spike-time dependent plasticity, coupled neural networks

## Abstract

Miniature robots are useful during disaster response and accessing remote or unsafe areas. They need to navigate uneven terrains without supervision and under severe resource constraints such as limited compute, storage and power budget. Event-based sensorimotor control in edge robotics has potential to enable fully autonomous and adaptive robot navigation systems capable of responding to environmental fluctuations by learning new types of motion and real-time decision making to avoid obstacles. This work presents a novel bio-inspired framework with a hierarchical control system to address these limitations, utilizing a tunable multi-layer neural network with a hardware-friendly Central Pattern Generator (CPG) as the core coordinator to govern the precise timing of periodic motion. Autonomous operation is managed by a Dynamic State Machine (DSM) at the top of the hierarchy, providing the necessary adaptability to handle environmental challenges such as obstacles or uneven terrain. The multi-layer neural network uses a nonlinear neuron model which employs mixed feedback at multiple timescales to produce rhythmic patterns of bursting events to control the motors. A comprehensive study of the architecture's building blocks is presented along with a detailed analysis of network equations. Finally, we demonstrate the proposed framework on the Petoi robot, which can autonomously learn walk and crawl gaits using supervised Spike-Time Dependent Plasticity (STDP) learning algorithm, transition between the learned gaits stored as new states, through the DSM for real-time obstacle avoidance. Measured results of the system performance are summarized and compared with other works to highlight our unique contributions.

## 1 Introduction

Biomimetic locomotion techniques draw inspiration from movement mechanisms exhibited by biological beings. The promised increase in robustness, adaptability, and efficiency of these techniques has led to considerable interest in robotics research with an increased focus on huge potential applications like rescue missions, hazardous environment exploration, assistance in domestic tasks, and healthcare (Wang et al., 2021). By emulating and utilizing strategies seen in nature, this approach inspires the design of robots to navigate various terrains and environments. A significant challenge that remains when working with this stratagem is achieving efficient and adaptive gait control through the integration of complex sensory inputs along with the generation of coordinated motor outputs.

**Figure 1:**
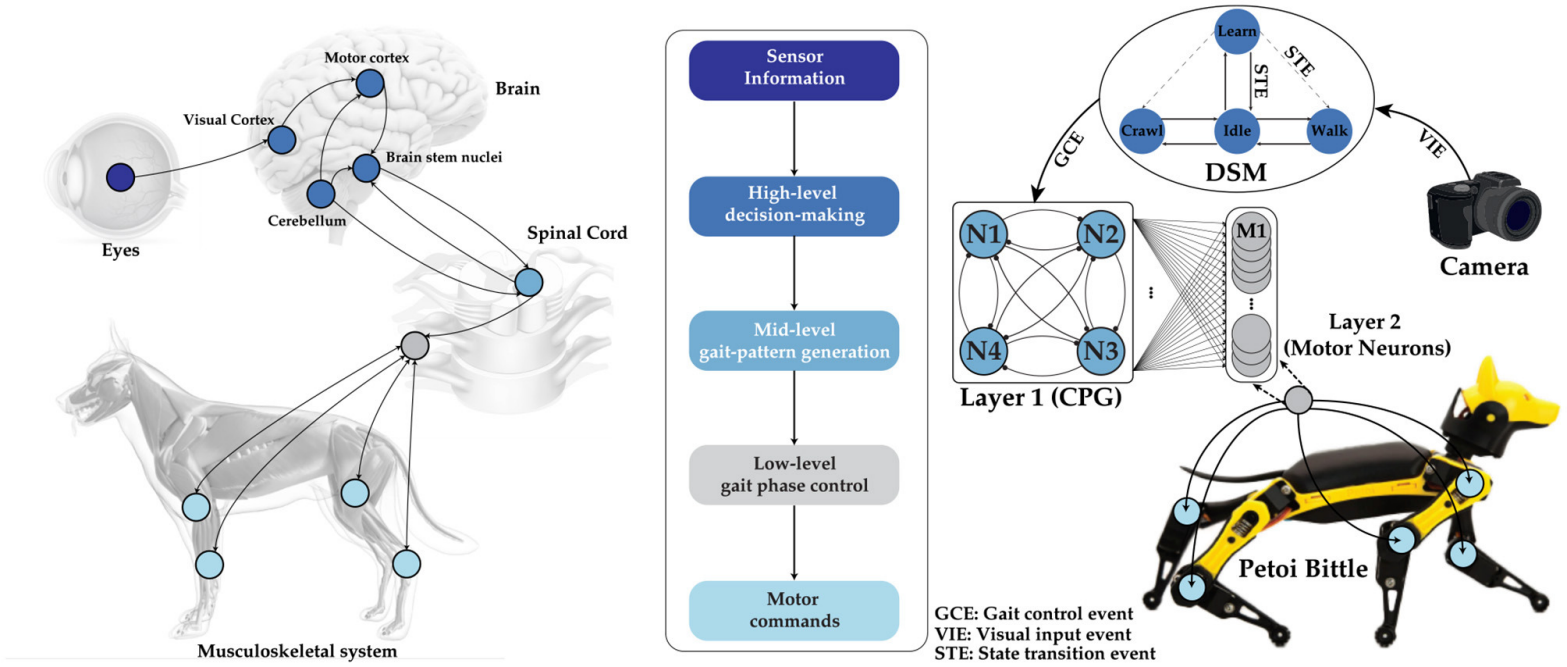
**(Left)** Schematic of biological information processing in a dog, from observation to actuation. The visual input received by the eye is processed in the brain, which then triggers the CPG located in the spinal cord to actuate the limbs for locomotion. **(Middle)** The flowchart of the hierarchical process in our implementation. **(Right)** A complete representation of our tunable multi-layer neural network, which includes a camera, a DSM, a bursting CPG as layer 1, and layer 2 for controlling motor neuron activations autonomosly. Image credits for the stock photo footage used in the preparation of this figure (Eye: ©reineg/Adobe Stock, Brain and Dog: ©Sebastian Kaulitzki/Adobe Stock, Spinal cord: ©RFBSIP/Adobe Stock, Camera: ©Janusz Lukaszewski/Istockphoto).

One promising solution to this challenge is utilizing Central Pattern Generators (CPGs) as described in Guertin (2009). CPGs are neural networks present in biological organisms that generate rhythmic patterns, crucial for controlling and coordinating locomotion. They gather sensory feedback from the environment to adjust locomotion patterns as needed. In animals, sensory inputs from systems like vision are processed by various brain regions, including the motor cortex, cerebellum, and brainstem nuclei, which then produce motor commands to activate the musculoskeletal system. The inherent capability of biological neurons to process discrete sensory events, rather than continuous data streams, enhances reaction time while maintaining the energy efficiency of the CPG. This efficiency has inspired many researchers to develop similar CPG networks to improve robot locomotion control, particularly in pedal-based robots, which mimic the structure of living organisms (Nakada et al., 2003, 2005; Simoni et al., 2004; Arena et al., 2005; Kier et al., 2006; Tenore et al., 2004; Song and Xu, 2022, 2023, 2024; Song et al., 2022). However, a significant challenge for such architectures is the dependence of the neural network on the choice of neuron model. CPGs leverage the diverse behaviors of biological neurons to create and adjust different rhythmic patterns (Izhikevich, 2006). In contrast, current Artificial Neural Networks (ANNs) typically overlook the nuanced behavior of neurons, using simplified activation functions that are generally adequate for most ANN applications (Buchanan, 2005; Szandała, 2021). This lack of attention is due to the complexity of implementing a Hodgkin–Huxley model (Hodgkin and Huxley, 1952), the most comprehensive neuron abstraction, in hardware, where the benefits are outweighed by the constraints of limited resources in robots. This paper explores the implementation of a CPG network to control the gaits of a quadruped robot, showcasing how this biologically inspired approach can enhance a robot's locomotion capabilities. The quadruped robot employs a hierarchical control system where high-level decision-making modules define the overall locomotion strategy. The mid-level CPG network generates rhythmic signals that control limb movements, while low-level controllers ensure precise joint actions. Sensors such as cameras provide real-time feedback, enabling the robot to adapt its gait to the terrain and maintain stability as shown in Figure 1.

A fully stand-alone autonomous (battery powered and no external inputs/wires) robot navigation system is realized in this work using three major novel contributions, (1) a tunable multi-layer neural network with a bursting CPG to generate different overlapping rhythmic gait patterns using a hardware-friendly numerical solver for nonlinear ODEs, (2) a dynamic state machine (DSM) that can adaptively grow as it learns new states and use the state transitions for real-time decision making in obstacle avoidance for robot navigation, and (3) a programmable synaptic weight matrix that can be trained using STDP to learn new gaits on command. Obstacle avoidance, in this context, refers to the robot's capability to detect an obstacle and adjust its path to prevent any collision.

The structure of the paper is as follows: Section 2 reviews the background of robotic gaits using CPG networks built with oscillators from related published articles and delves into the rationale behind the neuron selection for this work. Section 3 highlights the system design and novelty of this work by examining the CPG network architecture and analyses its behavior comprehensively. This section also discusses the DSM architecture in detail. Section 4 describes the hierarchical control system and the final neural network architecture and training. Section 5 presents the experimental results, and finally, Section 6 offers the conclusion.

## 2 Methods

### 2.1 Robot gaits

In biology, animals exhibit a variety of gaits such as walking, running, crawling, jumping, trotting etc. These gaits are achieved through precise control of the motor neurons in each limb with overlapping phases to ensure stability. The motor neurons at the knees and shoulders of each limb are activated multiple times in a single phase ensuring granular control of muscle flexion or extension that results in smooth locomotion.

**Figure 2:**
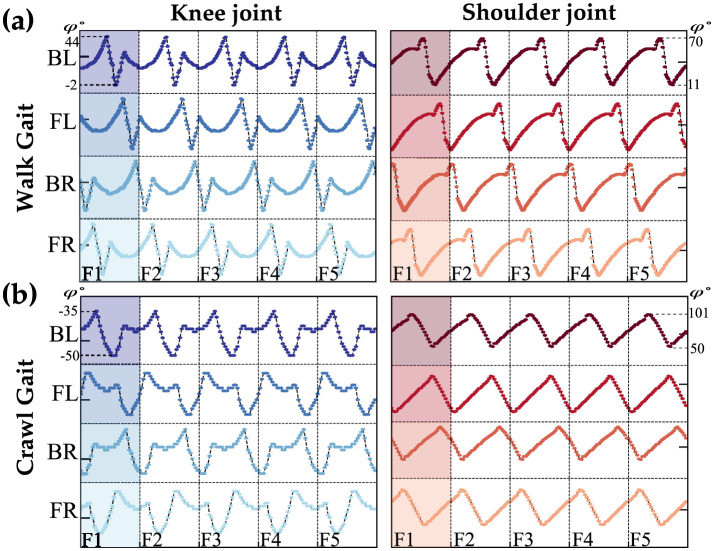
Waveforms represent the walk **(A)** and crawl **(B)** gaits for both knee and shoulder joints, as provided by the Petoi platform. The *y*-axis shows the angles, and each vertical line separates the frames. The labels BL, FL, BR, and FR stand for the back-left, front-left, back-right, and front-right legs of the Petoi Robot.

To enable the Petoi robot to learn and use different gaits for obstacle avoidance, it is necessary to study a few gaits such as the walk and crawl gaits. The quadruped robot consists of eight distinct joints that can be controlled independently, categorized into four shoulder and four knee joints. The walk and crawl gaits are illustrated in Figure 2. Each vertical line separates a frame, representing a collection of angles for one complete cycle of the gait. Figure 2 shows five frames for each condition, created by repeating the shaded area of each gait. The study of origin of the angles and ensuring stable locomotion for each gait is beyond the scope of this article; here, we focus on the choice of network architecture.

**Figure 3:**
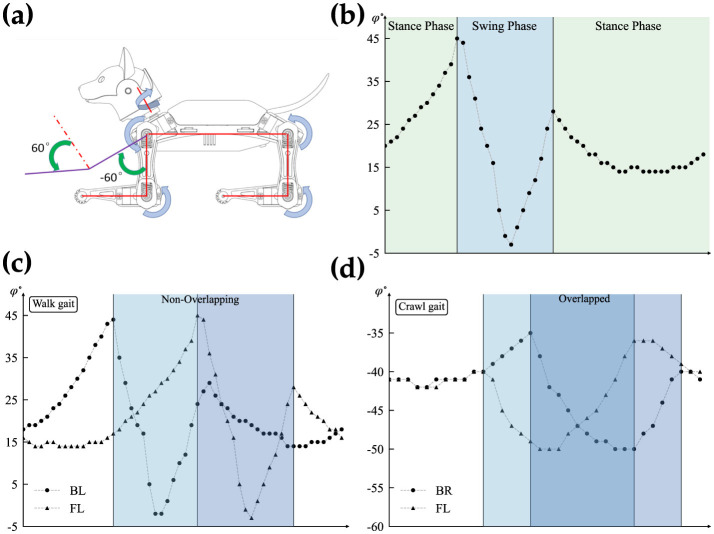
Detailed analysis of the overlapping gait patterns. **(A)** Shows reference coordinates for angle measurement (Petoi, 2024). **(B)** Illustrates the swing and stance phases. **(C)** Shows a non-overlapping swing phase, and **(D)** represents overlapping swing phases.

Within the walk/crawl, the angle profiles are almost identical but with a relative phase shift with respect to the reference joint (back left leg). For simplification, our qualitative observations categorize each frame into two regions: stance and swing phases, based on the rate of angle changes. The swing phase involves high changes, while the stance phase involves lower rate changes, as shown in Figure 3B. Figure 3A is showing the reference coordinate that is used to measure the angles. the detection of phases is qualitative approach and depends on the gait profile and should be calibrated separately. Further study of the gait reveals that swing phases either overlap by a certain percentage or do not overlap at all. Figures 3C, D highlights this behavior.

### 2.2 Spiking pattern generators

CPG networks have been implemented using spiking neurons such as the Leaky Integrate and Fire (LIF) neuron model. Previous works have demonstrated spiking CPGs to control robot locomotion (Lele et al., 2020, 2021; Vivekanand et al., 2023a). In these works, a network of LIF neurons that are bidirectionally coupled with synapses are tuned manually to generate different rhythmic patterns. However, the limitation of spiking CPGs is that a sequence of actions or motor commands must be generated with each spike. In other words, a single spike “event” has to correspond to a sequence of multiple motor command “events,” demanding the need for an external time-keeping signal, the digital clock. The granular control of the muscle movement found in animals is not achievable. This also defeats the purpose of an “event-based” system where the time to act has to be solely dictated by events, without the need for a timer clock.

Our previous work in Vivekanand et al. (2023b) addressed this issue by designing and implementing a bursting central pattern generator to control quadruped robots. Bursting CPGs allow for more granular control over the motion and speed of operation while retaining the ultra-low power and latency capabilities of spiking CPGs. However, the synaptic weights were manually tuned to produce different gaits.

In this work, a tunable bursting CPG network that can be trained using a supervised STDP algorithm is realized. The neuron chosen for this implementation is inspired by the model presented in Ribar and Sepulchre (2019) but highly optimized to run real-time on standard or specialized hardware, such as Raspberry Pi, with minimum resources while still retaining its bio-mimetic properties.

### 2.3 Nonlinear neuron model

The ability of CPGs to generate various patterns stems from the intricate temporal behavior of biological neurons. A key feature of biological neural networks is their remarkable ability to adapt and modulate their behavior across multiple scales, from ion channels controlling the spiking dynamics of individual neurons to larger brain regions governing higher-level cognitive functions like attention and learning. At the single neuron level, neuromodulators can precisely shape the firing patterns by modulating the collective conductance of different ion channels (Marder, 2012). This neuromodulation enables qualitatively different spiking regimes, such as the transition between tonic spiking and bursting oscillations, which is an essential mechanism for encoding sensory information in certain neural systems (Krahe and Gabbiani, 2004). Neuron abstraction such as the Hodgkin–Huxley model (Hodgkin and Huxley, 1952) or simplified models like FitzHugh-Nagumo (FitzHugh, 1961) and Izhikevich neurons (Izhikevich, 2003) for hardware implementation is restricted due to many factors. These approaches often involve fine parameter tuning and lack the robust neuromodulation properties observed in biological neurons (Indiveri et al., 2011; Van Pottelbergh et al., 2018). Maintaining the balance between biophysical realism and circuit complexity has been a major challenge (Indiveri et al., 2011).

**Figure 4:**
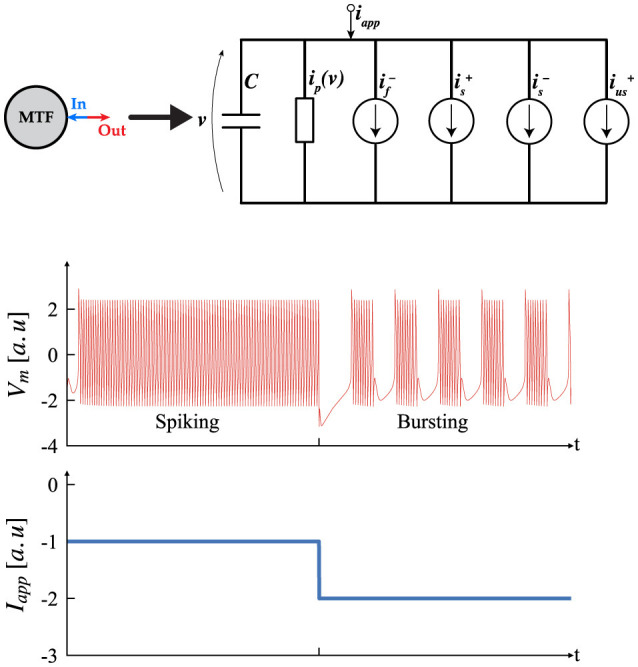
Circuit structure of the multi-timescale feedback, MTF, neuron which includes multiple conductance channels with varying time scales, similar to biological neurons. Transient simulation of the MTF neurons displaying Tonic Spiking and Tonic Bursting (Izhikevich, 2006), according to different *I*_*app*_. [a.u stands for arbitrary unit].

One promising alternative, introduced by Ribar and Sepulchre (2017), Ribar and Sepulchre (2019), and Liu et al. (2023), offers ease of hardware implementation and an intuitive analysis method, bypassing the need for complex bifurcation analysis. This approach features a simple neuron architecture inspired by biological neurons, as depicted in Figure 4, which includes multiple conductance channels operating on different time scales to naturally capture neuromodulation. This neuron model, referred to as the multi-timescale Feedback (MTF) neuron, leverages the input-output current-voltage (I–V) characteristics to provide a straightforward way to map neuron dynamics to the underlying ionic conductances.


$$\begin{array}{l} C\dot{V_{m}}=-(I_{p}^{+}(V_{m})+I_{f}^{-}(V_{f})+I_{s}^{+}(V_{s})+I_{s}^{-}(V_{s})\\ \;+I_{us}^{+}(V_{us})-I_{app}) \end{array}$$

(1) $$\begin{array}{l} \tau_{f}\overset{•}{V_{f}}=V_{m}-V_{f}\\ \tau_{s}\overset{•}{V_{s}}=V_{m}-V_{s}\\ \tau_{us}\overset{•}{V_{us}}=V_{m}-V_{us}\\ \left(\tau_{f}\ll \tau_{s}\ll \tau_{us}\right) \end{array}$$

The model illustrates the creation of spiking neurons by forming an excitable system (Sepulchre et al., 2019, 2018), which is achieved by interconnecting a passive membrane with fast negative and slow positive conductance elements. To replicate bursting behavior, the model requires an additional excitable system incorporating slow negative and ultra-slow positive conductance elements, thereby mimicking the conductance structures found in biological bursting neurons. Consequently, the MTF neuron can exhibit both tonic spiking and bursting behaviors, including the transition between these states (Izhikevich, 2006). The system's dynamic behavior can be described using a set of autonomous differential equations, as shown in Equation 1. In this context, $I_{p}^{+}$ represents a monotonic passive element with a positive slope, and $I_{x}^{\pm }=\alpha_{x}^{\pm }\tanh\left(V_{x}-\delta_{x}^{\pm }\right)$ describes the conductance channel (I–V) relationship. Here, the subscript *x* indicates the time scale, with (*x* = *f*) for fast, (*x* = *s*) for slow, and (*x* = *us*) for ultra-slow, while the superscript denotes the sign of the gain. Finally, *I*_*app*_ is an external current applied to the system to determine its operating regime.

In this context, the parameters α,  δ are referred to as the neuromodulatory parameters (NP). Once the NP and time constants are established, the operating regime of the system is controlled by *I*_*app*_. Figure 4 shows two possible outcomes and their transitions. It is important to note that spiking and bursting behaviors occur only within a specific range of *I*_*app*_; values outside this range will produce a constant stable output. This range can be determined using the input-output method described earlier. Determining the (NP), time constants, and the spiking/bursting range is beyond the scope of this article. For further details, interested readers should consult (Ribar and Sepulchre, 2019; Liu et al., 2023).

### 2.4 Half center oscillator network

The first step toward realizing CPG based on the MTF neuron involves understanding the behavior of the simplest structure, known as the Half-Center Oscillator (HCO) circuit. This circuit is formed by connecting two neurons via a synaptic connection, producing rhythmic, oscillatory signals similar to those of a CPG. Typically, a synaptic connection features a sigmoid activation function that can depend on any of the system's state variables, *V*_*x*_, where *x* denotes the time scale. The synaptic connection generates a current that is added to *I*_*app*_, resulting in a specific phase-locking behavior reminiscent of biological neurons. The ability of neurons to exhibit bursting behavior is a prerequisite for phase-locking behavior, underscoring the advantage of MTF neurons. The system of equations governing the HCO expands into an eight-dimensional system, with all state variables evolving together. It is important to note that the synaptic connection also depends on the state variable *V*_*x*_. Equation 2 provides the mathematical description of this system.


(2) $$\begin{array}{l} C\overset{•}{V_{m_{1}}}=-\left(I_{p}^{+}\left(V_{m_{1}}\right)+\sum \overset{\pm }{\underset{x}{I}}+\left(-I_{app}+I_{syn_{21}}\right)\right)\\ C\overset{•}{V_{m_{2}}}=-\left(I_{p}^{+}\left(V_{m_{2}}\right)+\sum \overset{\pm }{\underset{x}{I}}+\left(-I_{app}+I_{syn_{12}}\right)\right)\\ \;\tau_{f_{1}}\overset{•}{V_{f_{1}}}=V_{m_{1}}-V_{f_{1}}\\ \;\tau_{f_{2}}\overset{•}{V_{f_{2}}}=V_{m_{2}}-V_{f_{2}}\\ \;\tau_{s_{1}}\overset{•}{V_{s_{1}}}=V_{m_{1}}-V_{s_{2}}\\ \;\tau_{s_{2}}\overset{•}{V_{s_{2}}}=V_{m_{2}}-V_{s_{2}}\\ \;\tau_{us_{1}}\overset{•}{V_{us_{1}}}=V_{m_{1}}-V_{us_{1}}\\ \;\tau_{us_{2}}\overset{•}{V_{us_{2}}}=V_{m_{2}}-V_{us_{2}} \end{array}$$

Both neurons in HCO have identical NP and time constants and are preset in the Bursting mode by applying proper *I*_*app*_. In this context, subscript 1 refers to Neuron 1, and subscript 2 refers to Neuron 2 within an HCO. Equation 3


(3) $$\begin{array}{l} I_{x}^{\pm }=\alpha_{x}^{\pm }\tanh\left(V_{m}-\delta_{x}^{\pm }\right) \end{array}$$

denotes the general form of the conductance channel current for all time constant. The Equation 4 shows the synaptic current function where the *V*_*x*_ is state variable where the subscript *x* indicates the time scale, with (*x* = *f*) for fast, (*x* = *s*) for slow, and *x* = *us* for ultra-slow, while the superscript denotes the sign of the gain. The *B* is steepness factor, δ_*s*_ is Threshold and *W*_*ij*_ is the Strength of the connection.


(4) $$\begin{array}{l} I_{syn_{ij}}=\frac{W_{ij}}{1+e^{-B\left(V_{x}-\delta_{s}\right)}} \end{array}$$

**Figure 5:**
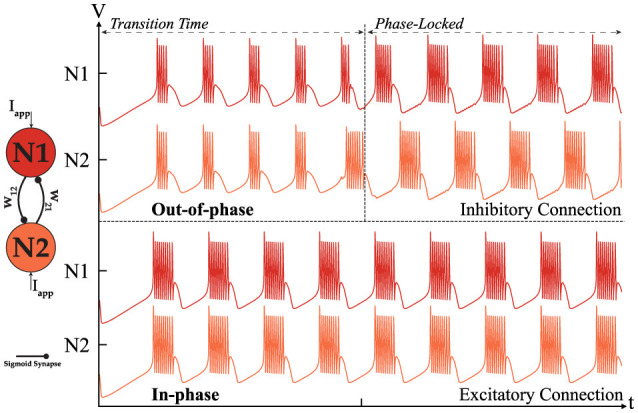
Simulation of two coupled neurons with excitatory or inhibitory connections showing two distinct modes of operation: out-of-phase **(top)** where *W* < 0. [|*w*_12_| = |*w*_21_| = 0.3] and in-phase **(bottom)** where *W* > 0.

Figure 5 depicts two potential behaviors of the HCO depending on the type of connection. An excitatory connection, where *W*_12_ = *W*_21_>0, results in an in-phase outcome, while an inhibitory connection, where *W*_12_ = *W*_21_ < 0, eventually produces an out-of-phase rhythm. Out-of-phase describes a state where the outputs of neurons are mutually exclusive, ensuring that no two neurons can produce bursts simultaneously. The primary difference between these modes is the transition phase required to achieve phase-locking when the connection is inhibitory. Our observations indicate that the strength of the connection influences the duration of the transition phase to some extent. If the connection is weak, the in-phase behavior of the HCO remains unaffected, while the inhibitory connection experiences a prolonged transition phase and eventually loses phase-locking entirely. Conversely, a strong connection can reduce the transition phase without negatively impacting the outcome, but as the connection strength increases, the spike profile begins to degrade, ultimately causing the HCO to lose phase-locking and enter a resting state. In summary, the in-phase mode is independent of the connection strength as long as (*I*_*app*_+*I*_*sy*_*n*__*ij*__) stays within the valid range needed for bursting mode. In contrast, the out-of-phase dynamic is directly influenced by the connection strength, as previously described. It is also important to note that the frequency for each neuron depend on *I*_*sy*_*n*__*ij*__(*W*_*ij*_) where the synaptic current is a function of the connection strength. Therefore, fluctuations of frequency is expected in the output. For more information on the dependency of the frequency on *I*_*syn*_, please refer to Ribar and Sepulchre (2019). The *i* denotes the pre-neuron while the *j* indicates the post-neuron. To facilitate the following analysis, it is useful to construct a weight matrix for the HCO. This approach simplifies and enhances the study of higher-order networks. Equation 5 presents such a matrix for the HCO.


(5) $$\begin{array}{l} G=\left[\begin{array}{cc} a_{11} & a_{12}\\ a_{21} & a_{22} \end{array}\right]=\left[\begin{array}{cc} 0 & w_{12}\\ w_{21} & 0 \end{array}\right] \end{array}$$

In all of our studies, the connection strengths *W*_*ij*_, whether inhibitory or excitatory, are equal unless stated otherwise. Therefore, for a network of size 2, *W*_12_ = *W*_21_ = *W*. The absence of self-connections for neurons means that the main diagonal of the weight matrix will always be zeros. Given these assumptions, the weight matrix for alternating and synchronous behavior is symmetrical. When *W*_*ij*_ < 0 it results in alternating oscillation, while *W*_*ij*_>0 leads to synchronous oscillation.

The origin of alternating and synchronous behavior in HCO has been extensively studied here (Wang and Rinzel, 1992). Wang et al. introduced two underlying mechanisms, “release” and “escape,” to explain out-of-phase oscillation. Although the neuron model used in Wang et al. original calculations is simplified, their findings can be extended to the MTF neuron since the underlying principles are based on neurons with multiple conductance channels and different time constants. For all practical purposes, qualitative observation of the temporal behavior of coupled neurons is sufficient to understand and evaluate the potential for forming a higher-order network suitable for robotic applications.

### 2.5 Central pattern generator network

**Figure 6:**
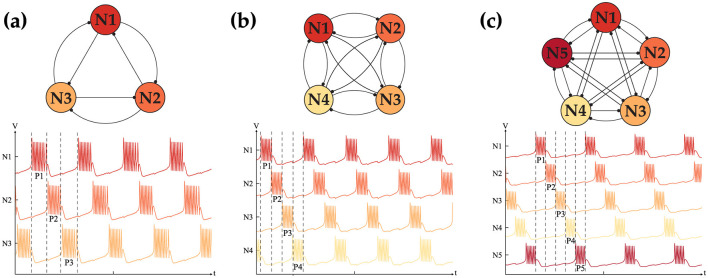
Out-of-phase dynamics of the CPG network with **(A)** *N* = 3, **(B)** *N* = 4, **(C)** *N* = 5 coupled neurons exhibiting 3, 4, 5 distinct phases, respectively.

To leverage the CPG as a controlling agent, forming a higher-order network is essential. Higher-order networks with similar synaptic connections are analyzed to assess their ability to exhibit behavior similar to that of the HCO. The results of our tests for different network sizes *N* are shown in Figure 6. Our observations confirmed that a fully interconnected network with *N* < 6 can generate in-phase and out-of-phase behavior similar to the HCO. However, as the network size increases, the ability to form alternating oscillations is lost, while synchronous oscillation remains unaffected. This behavior is due to the limited range of *I*_*tot*_ = (*I*_*app*_+*I*_*sy*_*n*__*ij*__) that can generate bursting. As the number of connections for each neuron increases, *I*_*tot*_ reaches its limit, preventing the network from sustaining the expected behavior. Figure 6 displays only the alternating oscillation, as the in-phase oscillation is evident. Forming the weight matrix for a given network size is straightforward and can be achieved by following the previously described rules. Equation 6 shows a generic weight matrix of size *N*:


(6) $$\begin{array}{l} G_{N}=\left[\begin{array}{ccccc} 0 & w_{12} & w_{13} & \cdots & w_{1N}\\ w_{21} & 0 & w_{23} & \cdots & w_{2N}\\ w_{31} & w_{32} & 0 & \cdots & w_{3N}\\ \vdots & \vdots & \vdots & \ddots & \vdots \\ w_{N1} & w_{N2} & w_{N3} & \cdots & 0 \end{array}\right] \end{array}$$

**Figure 7:**
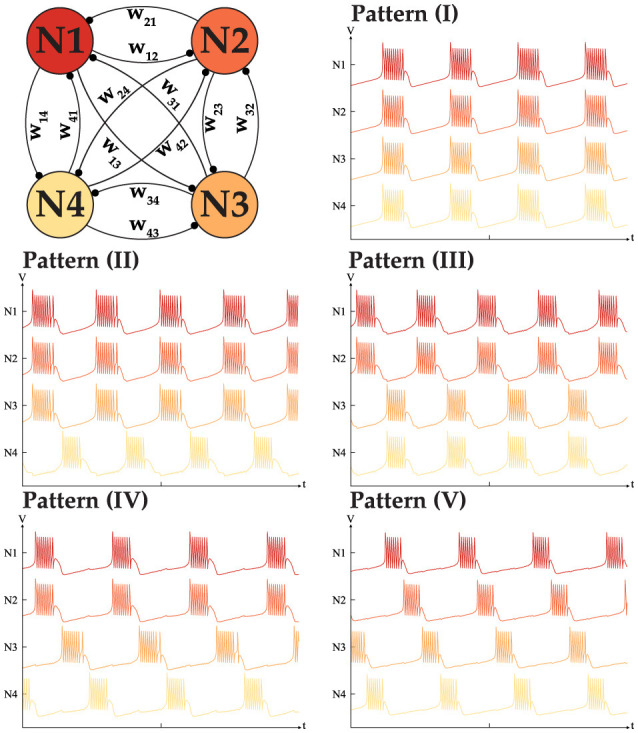
All five distinct phase patterns generated with a fully interconnected CPG network with *N* = 4 neurons.

To complete our analysis, the possibility of the generation of all the different patterns is studied. Assuming an ideal scenario where nothing limits the dynamical behavior of a network, our goal is to understand besides the two mentioned behaviors, can such a network generate all the possible intermediate patterns. For instance in a network of size *N* = 4, is a three sync neurons with one out-of-synch is a viable pattern or not. Following our hypothesis, for the network to generate such a pattern, it has to have excitatory connections within the in-phase neuron, while all the neurons should have inhibitory connections with the last neuron. To validate our assumption we conducted many simulations and were successful in recreating all the possible patterns with a fixed-sized network of *N* < 5. Figure 7 shows the all possibilities for the *N* = 4 with the weight matrix that is shown in Equation 7.


(7) $$\begin{array}{l} G_{4}=\left[\begin{array}{cccc} 0 & w_{12} & w_{13} & w_{14}\\ w_{21} & 0 & w_{23} & w_{24}\\ w_{31} & w_{32} & 0 & w_{34}\\ w_{41} & w_{42} & w_{43} & 0 \end{array}\right] \end{array}$$

To generate the patterns shown in Figure 7, the weight matrix should meet the following conditions, with *W*>0 in all cases.

- *w*_*ij*_ = *w*_*ji*_ = *W* for *i, j* ≤ 4.
- *w*_*i*4_ = *w*_4*i*_ = −*W* while *w*_*ij*_ = *w*_*ji*_ = *W* for *i, j* ≤ 3.
- *w*_12_ = *w*_21_ = *w*_34_ = *w*_43_ = *W* while the rest of the weights are −*W*.
- *w*_12_ = *w*_21_ = *W* while the rest of the weights are −*W*, with *w*_34_ = *w*_43_ = ρ*W*. where ρ>1.
- *w*_*ij*_ = *w*_*ji*_ = −*W* for *i, j* ≤ 4.

It is worth noting that in all the patterns, the weights of all the elements are the same. However, during our tests, it became clear that to generate pattern (IV), the connection *w*_34_ = *w*_43_ needs to be stronger to enforce phase locking between neurons 3 and 4. We assume that a stronger connection is required to push the neuron *I*_*tot*_ into the valid range necessary for generating the desired pattern. In summary, the total number of patterns for 2 ≤ *N* ≤ 5 is 2, 3, 5, and 7. While this idea can be generalized for *N* neurons our tests indicate that once *N*>5, the network behavior no longer meets expectations. This issue stems from limitations in the MTF model, particularly its restricted range which results in bursting behavior. Addressing this challenge remains an open question requiring further investigation and innovative solutions. Despite these limitations, the bursting CPG remains a promising candidate and can serve as a control agent, as demonstrated in our implementation for controlling a pedal-based robot. Details of our implementation will be discussed in the following section.

### 2.6 Numerical solver for nonlinear ODEs

Precise timing is crucial for creating an appropriate pattern for robotic applications. The objective of having a suitable numerical solver is to minimize or mitigate the solver's impact on the solution as much as possible. Achieving this level of accuracy relies on the chosen ODE numerical solver. As previously mentioned, the fundamentals of MTF neurons are based on having multiple time constants of different order. Consequently, accurately modeling such a system is a challenging task. Mathematically, the MTF neuron is represented by a stiff differential equation (Iserles, 2009). Stiff differential equations are a class of differential equations characterized by the presence of rapidly varying components that can cause significant numerical instability in standard explicit solution methods unless extremely small time steps are used. This rapid variation is often due to large negative real parts in the eigenvalues of the system's Jacobian matrix, leading to substantial disparities in the rates of change within the solution. As a result, explicit numerical methods like the Euler method can become impractically slow and computationally expensive. To address these challenges, implicit methods such as the backward Euler method, implicit Runge-Kutta methods, and stiffly stable methods like the backward differentiation formulas (BDF) are employed. To understand how different methods result in improvements, examining their differences in solving an ODE utilizing Euler method is helpful. Equation 8 presents the basic ODE and its simplification prior to the application of any numerical integration methods. The *t*_*N*_ = *t*_0_+*h* showing the next time step with *h* as increment, *y*(*t*_0_) = *y*_0_ is initial condition and next value is *y*(*t*_0_+*h*) = *y*_*N*_.


(8) $$\begin{array}{l} ẏ=f\left(t,y\left(t\right)\right)\Rightarrow dy=f\left(t,y\left(t\right)\right)dt\Rightarrow \\ \int_{t_{0}}^{t_{0}+h}y\left(t\right)dt=\int_{t_{0}}^{t_{0}+h}f\left(t,y\left(t\right)\right)dt \end{array}$$

Assuming the simple rectangle method (Leader, 2022) can result in two outcomes as it is shown in Equation 9a.


(9a) $$\begin{array}{l} y_{N}+y_{0}=f\left(t_{0},y\left(t_{0}\right)\right)\Delta t \end{array}$$

(9b) $$\begin{array}{l} y_{N}+y_{0}=f\left(t_{N},y\left(t_{N}\right)\right)\Delta t \end{array}$$

Equation 9a (a) illustrates the explicit Euler method, where the outcome of the next step relies solely on the initial condition. In contrast, part (b) depicts the implicit Euler method (Backward Euler method), where the next value cannot be directly determined. The implicit method requires an additional calculation step to obtain the value; however, this dependency prevents the next value from diverging and helps maintain the solution within a stable region. This is a crucial advantage when handling stiff systems of ODEs.

An additional improvement is to use multi-step integration (Iserles, 2009) instead of the simple rectangle method, which relies on only one previous value. This enhancement allows the solver to utilize more than one previous value, thereby capturing the full dynamical behavior of a system more accurately. Furthermore, fine-tuning the increment *h* could further enhance precision. The Backward Differentiation Formula (BDF) method incorporates both of these improvements and is our chosen approach for solving the system of stiff differential equations. The number of steps in a BDF solver is determined by the solver's order *s*. Here, we use the second-order BDF, taking into account the computational limitations of the platform (Pi) used for benchmarking our implementation. The s-order BDF formula is shown in the Equation 10 where *s* represents the order, *h* is the step size, and *a* and β are coefficients that depend on the order *s*.


(10) $$\begin{array}{l} \overset{s}{\underset{k=0}{\sum }}a_{k}y_{n+k}=h\beta f\left(t_{n+s},y_{t+s}\right) \end{array}$$

The coefficients can be easily found in numerous mathematical textbooks (Curtiss and Hirschfelder, 1952; Iserles, 2009). Assuming *s* = 2, the numerical relationship for the next unknown values can be expressed as shown in Equation 11.


(11) $$\begin{array}{l} \overset{2}{\underset{k=0}{\sum }}a_{k}y_{n+k}=h\beta f\left(t_{n+2},y_{t+2}\right)\Rightarrow \\ y_{n+2}-\frac{4}{3}y_{n+1}+\frac{1}{3}y_{n}=\frac{2}{3}hf\left(t_{n+2},y_{n+2}\right) \end{array}$$

Here the n is the indicator of timestep. The algorithm we used to implement the BDF solver for an N-dimensional system of equations is as follows.

- Constructing a system of ODEs based on the size of the network (N).
- Applying an alternative method to obtain additional initial conditions. In this case, the 1st-order Backward Euler method (BDF) is used to enhance the accuracy of the solution. While random initial conditions could be used, they do not ensure the convergence of the solution.
- Applying the 2nd-order BDF to the system's equations and solving for the unknown values.

Two important remarks are that no linearization is applied to the system of equations, and to solve the implicit function to obtain the unknown values, the Newton-Raphson method is used.

In Figures 4, 7 NP are the same as follows. The $-|\alpha_{f}^{-}|=|\alpha_{s}^{+}|=2$, $-|\alpha_{s}^{-}|=|\alpha_{us}^{+}|=1.5$ and $|\delta_{f}^{-}|=|\delta_{s}^{+}|=0$, $|\delta_{s}^{-}|=|\delta_{us}^{+}|=0$. The time constant are (τ_*f*_ = 1,  τ_*s*_ = 50,  τ_*us*_ = 2500). The synaptic threshold and steepness coefficient in all of the simulation was fixed at δ_*s*_ = −1 and *B* = 2. In Figure 5 the *I*_*app*_ = −1.5 for both neuron, Figures 6A, B the *I*_*app*_ = −1.6, Figure 7C the *I*_*app*_ = −1.8 and Figure 5 *I*_*app*_ = −1.85. The connection strength |*W*| for Figures 5, 6A, B is 0.3, for Figures 6C, 7 is 0.2 with ρ = 2. the sign is assigned based on connection type as described in dedicated sections earlier.

### 2.7 Dynamic state machine

**Figure 8:**
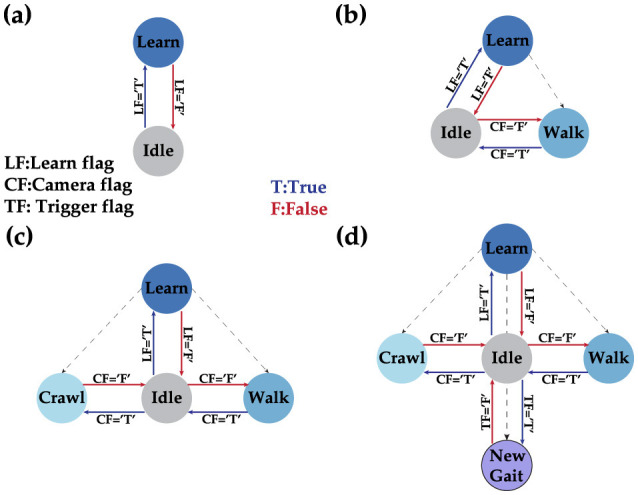
Different phases of the DSM where **(A)** shows the initial DSM when no gait is learned, **(B)** shows the situation after learning the walk gait, **(C)** shows the state after adding the crawl gait as a stable state, and finally **(D)** showcases the full DSM with the possibility to learn a new gait on demand.

In this work, a dynamic state machine (DSM) serves as the high-level organizer for our robotics system. The advantage of a DSM over a conventional finite state machine (FSM) is its flexibility to adapt to new states during operation without needing firmware reprogramming. Unlike the DSM, the FSM has a finite number of states and transitions, requiring a system reboot to implement changes. This dynamic behavior allows a robot to adapt to environmental changes by adjusting to new states as needed. The DSM prioritizes user input above all else when available. User input can include commands such as assigning next transition state or learning a new state. Learning new states involves learning a new gait pattern, which is defined as the sequence of motor angles that must be sent in a specific order for the robot to perform smooth locomotion actions. The initial state of the DSM includes only two states: idle and learn, as shown in Figure 8A. The robot remains in the idle state until a user command is issued, at which point it transitions to the learning state. The user command specifies the ideal gait matrix of the new gait, which the robot uses as a reference during the learning phase through the supervised STDP learning algorithm. Once the training is successful, the new state becomes a viable transition phase for the DSM. Figure 8B illustrates this for the walking gait. Although a single state is theoretically sufficient for autonomous control, our implementation of obstacle avoidance requires at least two states (walk and crawl) as shown in Figure 8C before starting its locomotion. Figure 8D shows our final implementation with the possibility of learning a new state if needed.

### 2.8 Hierarchical control system

**Figure 9:**
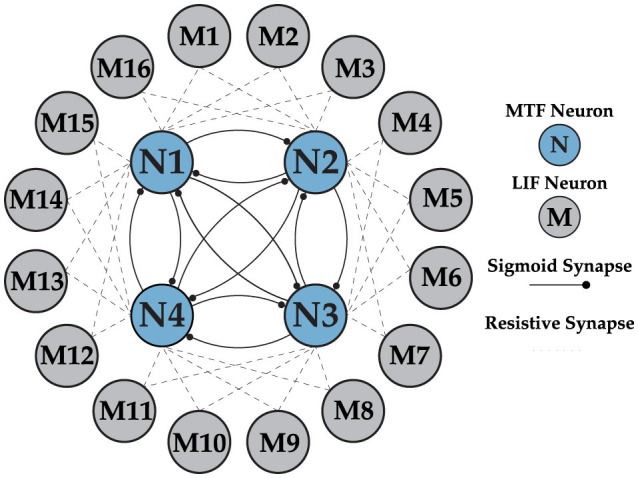
Multi-layer neural network architecture, implementing the Layer 1 (CPG) with MTF neurons and Layer 2 (motor neurons) with LIF neurons. There is a unidirectional connection from all the neurons in the CPG to the motor neurons, which is not shown in the figure for clarity.

Our control scheme is organized in hierarchical steps, as illustrated in the flowgraph in Figure 1. At the top layer, DSM oversees the entire network behavior, with user commands given the highest priority. The two-layer network consists of the CPG and a fully interconnected MTF neuron network without self-connections as layer one, and an integrate-and-fire (IF) neuron network as layer two. The layer two neurons are fully connected to the previous layers, as shown in the network architecture in Figure 9. The importance of the bi-layer structure will be explained later in this section in detail. This network is responsible for generating precise timing and patterns needed to send motor commands based on the gait. To demonstrate the benefits of our idea, we examine a simple obstacle avoidance scenario with two phases, using a camera event as the main controlling flag. Once the prerequisite states (walk and crawl) are learned, the system autonomously avoids horizontal obstacles in its path by transitioning from walking to crawling. It is worth noting that this approach can be extended to avoid any type of obstacle by adapting to a new state. However, as proof of concept, we focus solely on horizontal obstacles. This can be easily achieved by learning a new state for each type of avoidance.

### 2.9 Network architecture

Earlier analysis of the gaits is a key element in understanding the network structure shown in Figure 9 and optimizing the size of each layer. As explained earlier, the swing phases for different walk and crawl gaits have non-overlapping and overlapping characteristics, respectively. The non-overlapping parts of the timing sequence can be easily generated with the CPG, but forming overlapping sequences requires a new approach. The idea behind generating overlapping regions is simple and intuitive. Instead of comparing the individual outputs of layer one, a linear combination of multiple neurons is considered as the outcome when there is at least one common neuron. For instance, in the case of *N* = 4, *X*1 and *X*2 have 50% overlap.


(12) $$\begin{array}{l} X1=N1⊕N2\\ X2=N2⊕N3 \end{array}$$

It's worth noting that the ⊕ operation means *X*1 spikes whenever *N*1 or *N*2 spikes. This technique allows for generating any form of potential permutation of overlapping regions in an ideal scenario without restrictions. For *N* = 4, the limitation is that the overlap can only be either no overlap or 50% overlap. This is sufficient for our application, as the walk gait has no overlap, while the crawl gait has 50% overlap, as shown in Figure 3. Therefore, *N* = 4 is an optimal choice since it satisfies both requirements. The network behavior is not compromised as long as *N* < 6 and the combination of the 4 phases guarantees a possibility of 50% overlap. Furthermore, to generate a sufficiently long timing sequence to cover the stance phase, more neurons can be combined using the same ideas explained above.

To implement this technique, the second layer consist of 16 LIF neurons is used to ensure all the joints and distinct phases within each frame is covered. LIF model is opted to address the resource limitations of the testing platform (Pi) and to save energy. Additionally, using analog neurons for the secondary layer does not provide extra benefits. The IF neuron operates on a simple principle: the input is integrated until it reaches a predefined threshold *V*_*th*_, at which point the output generates a spike. The choice of threshold in our implementation is crucial and it is twofold:

- Determining the weight matrix strength.
- affecting the convergence time during the training.

Understanding the effect of the *V*_*th*_ on the weight matrix is straightforward. To have one-to-one correspondence in the spike generation between L1 and L2, the connection strength of *w*_11_ and *w*_12_ should be greater or equal to *V*_*th*_ and the rest of connection to layer 2 should be 0. This core idea can be used to set appropriate weights for the network to be able to generate walk and crawl gait.

Overall, both layers in the network collaborate to create an accurate timing sequence independent of the implementation platform clock (Pi in our test) to issue motor commands to the robot. The first layer, the CPG network, acts as the main coordinator due to its phase-locking capability and immunity to external influences, while the second layer shapes the pattern and fine-tunes the relative phase needed to precisely encode gait data for motor control as described previously. The CPG generates a 4-phase out-of-phase pattern, as shown in Figure 6B. The CPG layer is trained once and remains unchanged as long as needed. The second layer, however, is updated during learning phase. The general form of the weight matrix between layers 1 and 2 is shown in Equation 13 and will be updated via supervised STDP.


(13) $$\begin{array}{l} G_{NM}=\left[\begin{array}{ccccc} w_{11} & w_{12} & w_{13} & \cdots & w_{1M}\\ w_{21} & w_{22} & w_{23} & \cdots & w_{2M}\\ w_{31} & w_{32} & w_{33} & \cdots & w_{3M}\\ \vdots & \vdots & \vdots & \ddots & \vdots \\ w_{N1} & w_{N2} & w_{N3} & \cdots & w_{NM} \end{array}\right] \end{array}$$

### 2.10 Network training

The multi-layer network is trained using a supervised approach with the STDP algorithm. Training can be carried out offline. The optimal target synaptic weights are stored in internal memory and utilized during the training process. Training occurs when the Learning Flag (LF) is set to true through an operator command. STDP, a Hebbian learning process, is triggered by precise timing correlations between the spikes of neurons before and after synaptic connections, showing temporal asymmetry (Caporale and Dan, 2008; Taherkhani et al., 2020). It is believed to be fundamental to the processes of learning, information storage, as well as the development and refinement of neuronal circuits in the brain. STDP modifies the synaptic weights between neurons generally given by the following biphasic exponentially decaying function:


(14) $$\begin{array}{l} \Delta G_{syn}=\lambda sgn\left(\Delta t\right)e^{\frac{-sgn\left(\Delta t\right)\left(\Delta t\right)}{\tau_{learn}}} \end{array}$$

where Δ*t* = *t*_*pre*_−*t*_*post*_, λ is the learning rate parameter, τ_*learn*_ is time constant and *sgn* is sign function operator. If Δ*t* is >0, the postsynaptic neuron spikes after the presynaptic neuron, leading to long-term potentiation (LTP), whereas if the presynaptic neuron fires earlier than its postsynaptic counterpart, long-term depression (LTD) takes place. LTP results in an increase in the synaptic weight, whereas LTD results in a decrease. In this work, the training algorithm of both layers is summarized in the Algorithm 1. The learning algorithm works on the principle of achieving a desired gait pattern by iteratively adjusting synaptic weights, minimizing the error between the ideal gait pattern and the pattern obtained by applying the current weight input described in Algorithm 1. The convergence condition is that the spike times of the pre and post neurons must be within an error ϵ.

**Algorithm 1:**
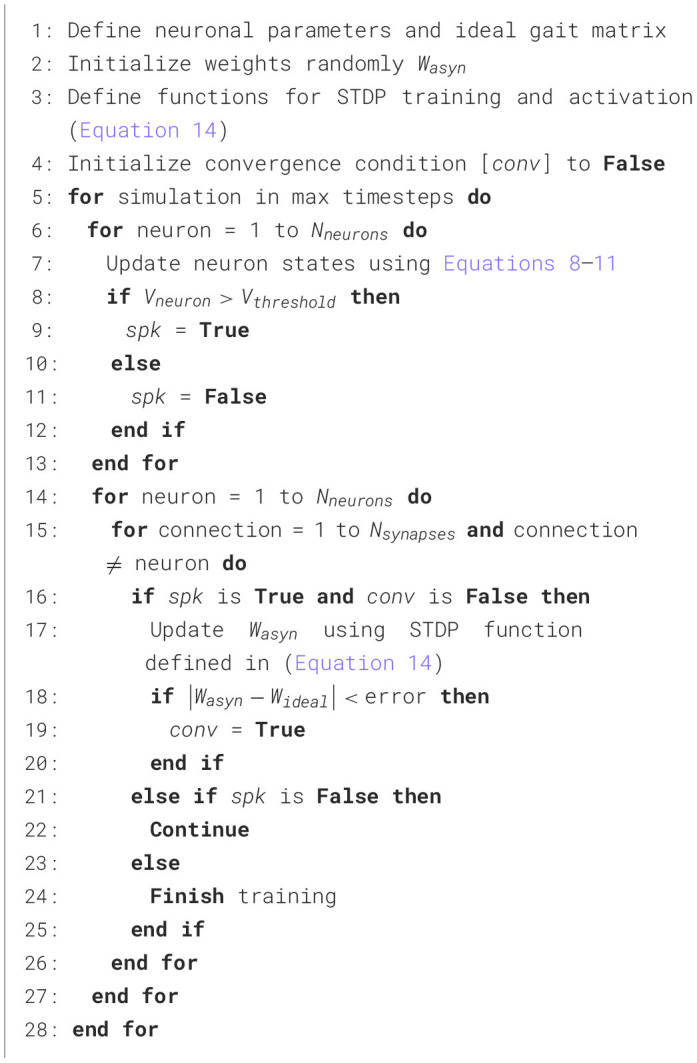
Learning gait patterns.

## 3 Results

**Figure 10:**
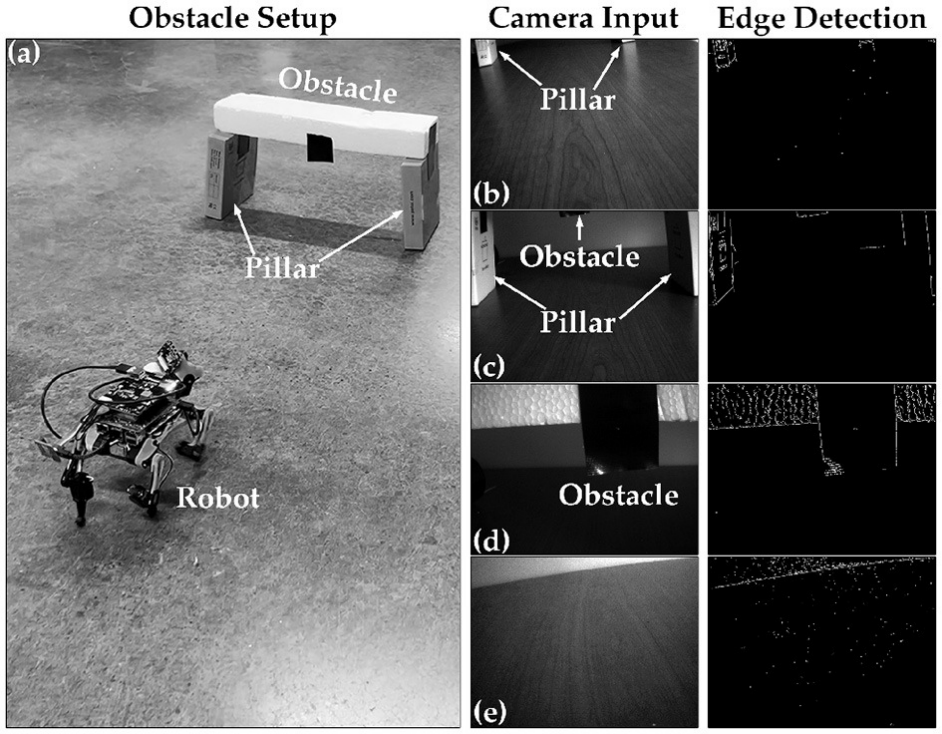
Obstacle avoidance demo setup. **(A)** Depict a rear view of the bot and obstacle setup. **(B)** Representing when the robot is far away from the obstacle, **(C)** showing when the robot approaches the obstacle, **(D)** the onset of the detecting object and initiating the avoidance process, and **(E)** showing when the robot avoided the obstacle successfully.

**Figure 11:**
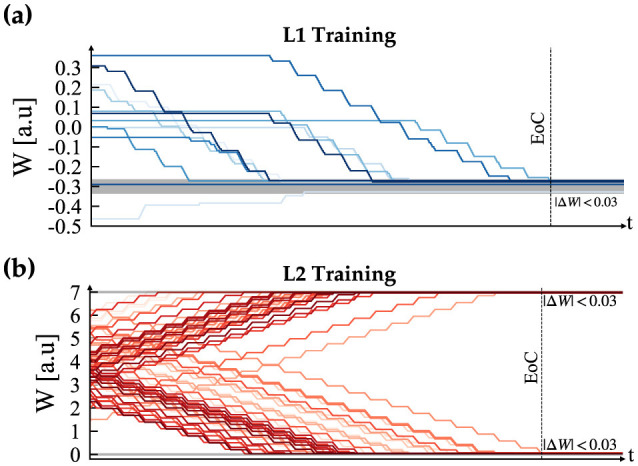
Supervised STDP training convergence plots, where **(A)** depicts the CPG training and **(B)** illustrates the result for the motor neurons. In both plots, EoC denotes End of Convergence.

Our proposed tunable multi-layer network is implemented in real-time on the Petoi Bittle robot, which comprises a Raspberry Pi to simulate the gait timing using bursting CPG and real-time decision making using DSM, and an Arduino as a servo controller. The use of multiple platforms is crucial because the Arduino resources are insufficient to handle complex simulations such as our network effectively. Additionally, to manage the complexity of solving a high-order system of ODEs, a parallel pipeline is used to distribute the workload across different processes, taking advantage of the multi-cores in the Pi platform. This is achieved using the “multiprocessing” Python library, which offers full control of the multi-process machine. Our implementation is divided into the following processes: camera, DSM, and our custom BDF solver. Each process occupies approximately (2.5%) of memory where the complete pipeline implemented on the Pi, took (6.35%) equivalent of (260.4 MB), where the average execution time of each time step was measured as 10 ms. Once the system boots for the first time, it waits for the learn flag. When the learn flag is received, it initiates the training of the CPG internal connections. After this step is completed, the robot is ready and will remain in the “Idle” state until the learning flag becomes true, as shown in Figure 8A. After training for all the gaits is completed, the system is ready to perform autonomous obstacle avoidance. Figure 10 shows the demonstration setup of the Petoi bot infront of an obstacle made of a bridge with two pillars. The task of the bot is to start walking and avoid the obstacle by detecting the bridge using the onboard camera and switching to crawl mode to cross underneath the bridge. Once the robot initiates walking, the camera captures footage at a relatively slower frame rate, calibrated based on the robot's movement speed. The captured footage is then processed using the Canny edge detection algorithm in the OpenCV library, as shown in Figures 10B–E. The result generates the camera flag event, which triggers the transition between states in autonomous mode. If the camera detects an object close to the robot while walking (camera flag CF = True), the robot will transition to the “Idle” state and then to the “Crawl” state. The training results of each layer's training are shown in Figure 11.

**Figure 12:**
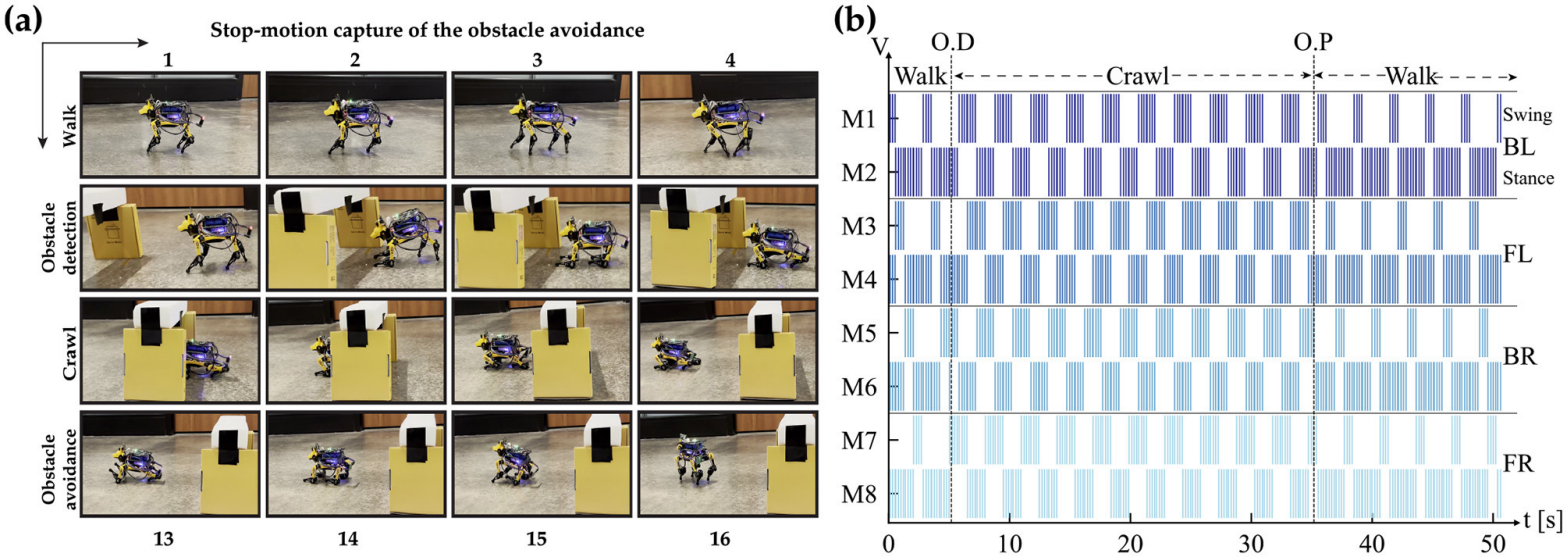
**(A)** A frame-by-frame capture of the obstacle avoidance process. **(B)** Illustrates the output of layer 2 (motor neurons) controlling the knee joints (Shoulder joints have the same identical patterns). O.D. and O.P. denote Obstacle Detection and Obstacle Passed, respectively. A demonstration video of the process is available at https://youtu.be/4E66LwjxBy4.

The CPG is always in free-run mode to avoid the initial settling time needed to enter the phase-locking mode. Layer 2 then combines the bursting spikes of Layer 1 CPG output to generate the timing needed for each motor control sequence. Finally, the motor command is sent out according to the generated sequence of spike events. This method creates a jitter-tolerant sequence, thanks to the CPG, which is independent of the digital clock of the system. The stop motion capture of the obstacle avoidance demo and the raster plot of bursting spike events generated during the entire duration are presented in Figure 12. The figure highlights the initial walk phase until Obstacle Detection (OD), followed by the transition to the crawl phase until passing the obstacle. A video demonstration of the robot showing all the different patterns can be accessed via the following link “https://youtu.be/4E66LwjxBy4.”

We compared our work with the most similar literature, summarized in Table 1. To the best of our knowledge, our work is the only one utilizing a hardware-friendly bursting CPG with an adaptive DSM to address different demands arising from deploying a robot in various environments, unlike the conventional finite state machine (FSM) used in other works. Additionally, our custom nonlinear ODE solver can be easily implemented on any platform. Overall, our proposed framework offers a flexible robot navigation system that minimizes the usage of the resources of the implementation platform.

**Table 1:** Comparison of CPG based systems for robot navigation.

**Robot**	**Features**	**Training algorithm**	**Sensory input**	**Task**	**Latency per event (ms)**	**Memory (MB)**	**Energy (J)**
Quadruped (Aljalbout et al., 2020)	Spiking CPG	Remote supervised method (Ponulak, 2005)	–	Single gait	–	–	–
Quadruped (Gutierrez-Galan et al., 2020)	Spiking CPG	Manual design	–	Multiple gaits	0.00346	–	–
Hexapod (Lele et al., 2020)	Spiking CPG	Stochastic reward	Gyro + camera	Multiple gaits	–	–	855.1n*
Hexapod (Lele et al., 2021)	Spiking CPG	Supervised	DVS Camera	Object approach	–	–	2.5m**
Quadruped (Vivekanand et al., 2023b)	Bursting CPG	Manual design	–	Multiple gaits	3.54	–	–
**Quadruped this work**	**Bursting CPG, Adaptive DSM, MTF neurons, BDF solver**	**Supervised STDP**	**Camera Events**	**Obstacle avoidance**	**10**	**260.4**	68m***

*,** In both cases, energy consumption per event/spike is reported as if the Loihi platform is used, but it appears that it is not actually utilized to control the robot. As a result, the actual energy consumption is unavailable. Only the CPG network's energy consumption is provided, while the energy use of the peripheral system is not accounted for.

*** This represents the energy consumption per event, including the peripheral system (Raspberry Pi), for the entire obstacle avoidance test (≈ 50 s).

## 4 Discussion

This work presents a bio-inspired framework for event-based sensorimotor control to enable autonomous robot navigation. The framework consists of a tunable multi-layer neural network with a CPG to generate and govern timing of motion, a DSM for learning new gaits, switching gaits based on input stimulus and a training algorithm based on STDP to determine the synaptic weights of the multi-layer neural network. The CPG can be trained to generate different overlapping rhythmic gait patterns using bursting neurons simulated with a hardware-friendly numerical solver for nonlinear ODEs. The DSM can adaptively grow as it learns gait patterns as new states and uses the state transitions triggered by sensory events (camera input) for switching gaits real-time for obstacle avoidance. A detailed analysis of bursting CPG networks, built with MTF neurons and simulated with a hardware-friendly BDF solver, is presented and limitations discussed. The entire framework is implemented on the Raspberry Pi hardware platform housed on a battery powered Petoi robot that uses Arduino platform to control eight servo motors present in the quadruped knees and shoulders. Neural network training results for the bursting CPG (Layer 1) and Motor neurons (Layer 2) using the STDP algorithm to learn the walk and crawl gaits are shown. A fully autonmous and standalone demonstration of the Petoi robot navigating and avoiding an obstacle is presented. Measured results of memory usage and execution time are summarized. The results demonstrate the potential of the proposed event-based framework in enabling fully autonomous navigation in edge robotics.

## Data Availability

The original contributions presented in the study are included in the article/supplementary material, further inquiries can be directed to the corresponding author.
